# Correlation between antimicrobial resistance and biofilm formation capability among *Klebsiella pneumoniae* strains isolated from hospitalized patients in Iran

**DOI:** 10.1186/s12941-021-00418-x

**Published:** 2021-02-15

**Authors:** Shadi Shadkam, Hamid Reza Goli, Bahman Mirzaei, Mehrdad Gholami, Mohammad Ahanjan

**Affiliations:** 1 Department of Microbiology and Virology, Faculty of Medicine, Mazandaran University of Medical Sciences, Sari, Iran; 2 Antimicrobial Resistance Research Center, Mazandaran University of Medical Sciences, Sari, Iran

**Keywords:** *Klebsiella pneumoniae*, Biofilm, Antimicrobial resistance, MDR, XDR

## Abstract

**Background:**

*Klebsiella pneumoniae* is a common cause of nosocomial infections. Antibiotic resistance and ability to form biofilm, as two key virulence factors of *K. pneumoniae*, are involved in the persistence of infections. The purpose of this study was to investigate the correlation between antimicrobial resistance and biofilm formation capability among *K. pneumoniae* strains isolated from hospitalized patients in Iran.

**Methods:**

Over a 10-month period, a total of 100 non-duplicate *K. pneumoniae* strains were collected. Antibiotic susceptibility was determined by Kirby–Bauer disk diffusion method according to CLSI. Biofilm production was assessed by tissue culture plate method. Finally, polymerase chain reaction was conducted to detect four families of carbapenemase: *bla*_IMP_, *bla*_VIM_, *bla*_NDM_, *bla*_OXA−48_; biofilm formation associated genes: *treC*, *wza*, *luxS*; and *K. pneumoniae* confirming gene: *rpoB.*

**Results:**

Most of the isolates were resistant to trimethoprim-sulfamethoxazole (52 %), cefotaxime (51 %), cefepime (43 %), and ceftriaxone (43 %). Among all the 100 isolates, 67 were multidrug-resistant (MDR), and 11 were extensively drug-resistant (XDR). The prevalence of the *bla*_VIM_, *bla*_IMP_, *bla*_NDM_, and *bla*_OXA−48_ genes were 7 , 11 , 5 , and 28 %, respectively. The results of biofilm formation in the tissue culture plate assay indicated that 75 (75 %) strains could produce biofilm and only 25 (25 %) isolates were not able to form biofilm. Among these isolates, 25 % formed fully established biofilms, 19 % were categorized as moderately biofilm-producing, 31 % formed weak biofilms, and 25 % were non-biofilm-producers. The antimicrobial resistance among biofilm former strains was found to be significantly higher than that of non-biofilm former strains (*p* < 0.05). Molecular distribution of biofilm formation genes revealed that 98 , 96 , and 34 % of the isolates carried *luxS*, *treC*, and *wza* genes, respectively.

**Conclusions:**

The rise of antibiotic resistance among biofilm-producer strains demonstrates a serious concern about limited treatment options in the hospital settings. All of the data suggest that fundamental actions and introduction of novel strategies for controlling of *K. pneumoniae* biofilm-related infections is essential.

## Background

*Klebsiella pneumoniae* has been clinically identified as one of the most important opportunistic pathogens responsible for nosocomial infections or healthcare-associated infections, including septicemia, urinary tract infections, soft tissue infections, and pneumonia [1]. Today the increasing rate of drug resistance among *K. pneumoniae* isolates is a main concern worldwide [2]. Multidrug-resistant *K. pneumoniae* (MDR-*Kp*), which is resistant to many commonly used antibiotic classes such as aminoglycosides, fluoroquinolones, cephalosporins, and carbapenems, has been increasingly reported from Iran [3]. MDR-*Kp* is a subject of great concern as it not only causes severe and fatal disease, but also increases the length of hospitalization, resulting in increased treatment charges [4]. Carbapenems are a class of highly effective antibiotic agents versus infections caused by MDR-*Kp* strains, though their application in administration of infections is threatened by development of carbapenem-resistant *K. pneumoniae* (CR-*Kp*) strains [3, 5]. CR-*Kp* strains are produced in response to a combination of one or more of the following mechanisms: stable derepression of AmpC, efflux pump overexpression, low outer membrane permeability, altered penicillin-binding proteins (PBPs), or production of class B metallo-β-lactamases (MBLs) and carbapenem-hydrolysing class D oxacillinases [6, 7]. Many *K. pneumoniae* are able to form biofilms, where bacteria are surrounded in an extracellular polysaccharide (EPS) matrix, that results in increased antibiotic impermeability [8, 9]. Formation of a biofilm also protects the bacteria from being eliminated by phagocytic cells [10]. The resulting resistance to antimicrobials from biofilm formation has been shown to hamper therapy [11]. Some virulence-related genes, including the cps gene cluster (a capsule encoding gene), *mrk* (type III fimbriae), *wbbM*, and *wzm* (LPS-synthesis-related genes) are involved in the biofilm production [12]. In addition, LuxS (type II quorum-sensing regulatory system) and *pgaABCD* operon, which are responsible for synthesis of poly-β-1,6-*N*-acetyl-d-glucosamine (PGA) (PgaC and PgaD) and secretion of PgaA and PgaB adhesions, which affect biofilm development by increasing cell-to-cell interactions as well as abiotic surface binding and intercellular adhesion [11]. Though, it seems that antimicrobial resistance and bacterial tendency to biofilm production, play a key role in the emergence of MDR-*Kp* strains, the clear correlation between these traits has not been completely elucidated. Thus, the purpose of this study was to investigate the antimicrobial resistance and biofilm formation capability among *K. pneumoniae* strains isolated from hospitalized patients.

## Methods

### Sampling and bacterial isolation

During a ten-month period from April 2016 to January 2017, this cross-sectional study was performed on hospitalized patients referred to four educational teaching hospitals in Sari, North of Iran. Microbial isolates were initially identified using conventional tests, including Gram staining, indole production, motility, lactose fermentation, hydrogen sulfide (H_2_S) production, citrate and urease test, lysine decarboxylase and MR-VP and subsequently, confirmed by API20E (bioMerieux, France) [3]. Species identification was confirmed by *rpoB* gene PCR. Each *K. pneumoniae* isolate was preserved in Trypticase Soy Broth (TSB) (Merck Darmstadt, Germany) with 20 % glycerol at − 70 °C.

### Antimicrobial susceptibility testing

According to the clinical and laboratory standards Institute protocol (CLSI; M100-S14) [13], the antibiotic susceptibility was tested by disk agar diffusion method on the Mueller-Hinton agar plates (MHA) (Merck, Darmstadt, Germany) for ceftazidime (CAZ: 30 µg), cefotaxime (CTX: 30 µg), imipenem (IPM: 10 µg), meropenem (MEM: 10 µg), ciprofloxacin (CIP: 5 µg), cefepime (FEP: 30 µg), ceftriaxone (CRO: 30 µg), amikacin (AN: 30 µg), gentamicin (GM; 10 µg), and trimethoprim-sulfamethoxazole (SXT; 5 µg) (MAST Diagnostics, Merseyside, UK).

Strains non-susceptible to at least three or more antimicrobial classes were defined as MDR, and those non-susceptible to at least one agent in all but two or more antimicrobial categories were considered as possible XDR, and the strains that non-susceptibility to all agents in all antimicrobial categories were defined as possible pan drug-resistant (PDR) [14]. *Escherichia coli* ATCC 25922 was used as control organism in susceptibility testing.

### Phenotypic detection of MBLs

CR-*Kp* strains were assessed for MBL production using the double‑disk synergy test (DDST). Briefly, a 0.5 McFarland turbidity (1.5 × 10^8^ CFU/mL) of the microbial suspension was plated on MHA. Then, two 10-µg IPM disks were located on the MHA plates. Following this, 10 µL of MBL inhibitor (iMBL; 0.5 M EDTA) was directly added to one of the disks to reach a desired concentration of 750 mg. After an overnight incubation period at 37 °C, inhibition zone diameter (IZD) of all disks was recorded and compared. The strains were recognized as an MBL-producing isolate when the difference in the IZD was ≥ 7 mm [15].

### Quantitative biofilm production assay

Tissue culture plate (TCP) assay was used for quantitative measurement of biofilm production in *K. pneumoniae* isolates. For each isolate, several colonies were inoculated in 10 mL of TSB with 1 % glucose (TSBG) and incubated for 24 h at 37 °C in stationary phase; after incubation, each suspension (adjusted to 0.5 McFarland (~ 1.5 × 10^8^ CFU/mL) was diluted 1:100 in fresh TSB. Each wells of sterile 96-well microtiter plates (Sigma-Aldrich, USA) were filled with 200 µl of microbial suspension. As the negative control, sterile TSBG was employed, while the *K. pneumoniae* ATCC 700603 was used as the strong biofilm producer positive control. The wells were washed four times with 0.2 mL of phosphate buffer saline (PBS, pH 7.2), desiccated for 1 h at 60 °C and stained for 15 min with 180 µl of 2 % Hucker’s crystal violet (0.1 % w/v). Additional stain was rinsed off with sterile distilled water. The dye bound to the adherent cells was solubilized with 180 µl of 33 % (v/v) glacial acetic acid (Zorka Pharma, Sabac, Serbia) per well and the absorbance was measured at 570 nm. Each assay was performed in triplicate, and repeated four times. The optical density cut-off (ODc) was declared as three standard deviations above the mean OD of the negative control. Biofilm formation was recorded as follows: non-biofilm forming (A_570_ < 1); +, weak (1 < A_570_ < 2); ++, moderate (2 < A_570_ < 3); +++, strong (A_570_ > 3) [16].

### Molecular examination

PCR was utilized for detection of three common types of MBL genes (*bla*_IMP_, *bla*_VIM_, *bla*_NDM)_ and class D oxacillinase (*bla*_OXA− 48_) as carbapenemases genes, biofilm-encoding genes (*treC*, *wza* and *luxS*) and *K. pneumoniae* confirming gene (*rpoB*). Bacterial DNA template was obtained from the purified colonies grown on the brain heart infusion agar plates (Merck, Darmstadt, Germany) using a bacterial genomic DNA extraction kit (Bioneer, Daejeon, Korea), and then kept at −20 °C. The oligonucleotide primer sequences used in the present work are shown in Table 1 [11, 16, 17]. The PCR reaction was performed in a final volume of 25 µL, including the following ingredients: 1.0 µL of extracted DNA, 12.5 µL Maxima Hot Start PCR Master Mix (2X) (Thermo, Waltham, Massachusetts, United States), 0.8 µL of each primer, and 10.4 µL of sterile distilled water. The samples were amplified in thermal cycler (Eppendorf, Hamburg, Germany) as follows: initial denaturation at 94 °C for 7 min, followed by 33 cycles of denaturation at 95 °C for 30 s, annealing at 53 °C for 60 s, extension at 72 °C for 60 s and a final extension at 72 °C for 5 min. PCR products were electrophoresed on 1.5 % agarose gel stained with Gel Red™ (Biotium, Landing Pkwy, Fremont, CA, USA), then photographed under an ultraviolet transilluminator (Bio-Rad, Hercules, USA). Both positive and negative controls were used in PCR for optimization and standardizing of molecular test.

**Table 1:** List of primers used for PCR amplification

Target gene	Primer sequences (5′ → 3′)	Product size (bp)
*rpoB*	5ʹ- CAACGGTGTGGTTACTGACG-3ʹ 5ʹ- TCTACGAAGTGGCCGTTTTC-3ʹ	108
*bla*_IMP_	5ʹ- TGAGCAAGTTATCTGTATTC -3ʹ 5ʹ- TTAGTTGCTTGGTTTTGATG -3ʹ	740
*bla*_VIM_	5ʹ- GATGGTGTTTGGTCGCATA-3ʹ 5ʹ- CGAATGCGCAGCACCAG-3ʹ	390
*bla*_NDM_	5ʹ- GGTTTGGCGATCTGGTTTTC-3ʹ 5ʹ- CGGAATGGCTCATCACGATC-3ʹ	621
*bla*_OXA-48_	5ʹ- TTGGTGGCATCGATTATCGG-3ʹ 5ʹ- GAGCACTTCTTTTGTGATGGC-3ʹ	428
*treC*	5ʹ- CCGACAGCGGGCAGTATT -3ʹ 5ʹ- CGCCGGATTCTCCCAGTT-3ʹ	71
*wza*	5ʹ- CGACAGTGAATGCGTCATT-3ʹ 5ʹ- TGACTGGCTTCCTGATGC-3ʹ	309
*luxS*	5′- GCCGTTGTTAGATAGTTTCACAG -3' 5′- CAGTTCGTCGTTGCTGTTGATG -3'	447

### Data analysis

SPSS software version 16 for windows (SPSS Inc., Chicago, IL, USA) was used for statistical analysis. Statistical significance in this study was *P* < 0.05.

## Results

### Bacterial isolation

In total, 100 non-duplicative clinically-relevant *K. pneumoniae* were collected from urine (n = 61), wound exudates (n = 13), intratracheal tube (ITT) (n = 11) blood (n = 9), and sputum (n = 6). The prevalence of isolates in hospital wards was as follow: intensive care units (ICUs) (n = 31), burn (n = 24), dialysis (n = 16), hematology-oncology (n = 9), respiratory care (n = 7), internal medicine (n = 6), neonatal intensive care unit (NICU) (n = 5), and surgery (n = 2). It worth mentioning that the positive culture in urine specimens was defined as a growth of more than 10^5^ CFU/mL. The mean age of the patients was 49.6 years (range, 18 to 86 years), where 60 % (n = 60) patients were female and 40 % (n = 40) were male.

### Antimicrobial resistance profile

Based on the acquired antibiotic resistance pattern, all *K. pneumoniae* strains were considered resistant to at least one of the tested antibiotics. In detail, 67 % of the isolates resulted resistant to three or more antimicrobials, and 11 % isolates were classified as XDR. No isolate was identified as PDR. As presented in Table 2, most of the isolates were resistant to SXT (n = 52/100, 52 %), CTX (n = 51/100, 51 %), FEP (n = 43/100, 43 %), and CRO (n = 43/100, 43 %). Further, the lowest resistance rate was related to the AN (n = 7/100, 7 %), all of which strains were also were MDR-*Kp*. On the other hand, all non-MDR*-Kp* isolates were susceptible to AN. Of the 35 IPM-resistant *K. pneumonia* isolates, 74.3 % (n = 26) were MBL-positive with the DDST, all of which were MDR.

**Table 2:** Antimicrobial susceptibility profile of *K. pneumoniae* isolates

Susceptibility profile		SXT	CTX	FEP	AN	IPM	MEM	CRO	GM	CAZ	CIP
MDR	S	25 (37.3%)	26 (38.8%)	27 (40.3%)	55 (82.1%)	41 (61.2%)	38 (56.7%)	29 (43.3%)	48 (71.6%)	28 (41.7%)	19 (28.3%)
	I	4 (5.9%)	8 (11.9%)	5 (7.5%)	5 (7.5%)	2 (2.9%)	1 (1.5%)	9 (13.4%)	2 (2.9%)	8 (11.9%)	4 (5.9%)
	R	38 (56.7%)	33 (49.3%)	35 (52.2%)	7 (10.4%)	24 (35.8%)	28 (41.7%)	29 (43.3%)	17 (25.4%)	31 (46.3%)	44 (65.6%)
Non-MDR	S	18 (54.5%)	22 (66.6%)	22 (66.6%)	31 (93.9%)	22 (66.6%)	23 (69.6%)	16 (48.5%)	25 (75.5%)	18 (54.5%)	19 (57.5%)
	I	1 (3%)	4 (12.1%)	3 (9.1%)	2 (6.1%)	0 (0.0%)	1 (3%)	3 (9.1%)	0 (0.0%)	2 (6.1%)	5 (15.2%)
	R	14 (42.4%)	18 (54.5%)	8 (24.2%)	0 (0.0%)	11 (33.3%)	9 (27.3%)	14 (42.4%)	8 (24.2%)	13 (39.4%)	9 (27.3%)

*MDR* multidrug resistant, *S* susceptible, *I* intermediate resistant, *R* resistant, *SXT* Trimethoprim-sulfamethoxazole, *CTX* cefotaxime, *FEP* cefepime, *AN* amikacin, *IPM* imipenem, *MEM* meropenem, *CRO* ceftriaxone, *GM* gentamicin, *CAZ* ceftazidime, and *CIP* ciprofloxacin

### Biofilm production

Our data revealed that 75 % of *K. pneumoniae* isolates were biofilm-producers. In this study, 31 , 25 , and 19 % of isolates were weakly, strongly, and moderately biofilm-producing strains, respectively. In addition, 25 % of the isolates were considered non-biofilm producers. The prevalence of biofilm formation in MDR isolates was significantly higher than in non-MDR isolates (p < 0.05) (51 % compared to 24 %) (Table 3). The biofilm-formation ability among the isolates collected from sputum was significantly higher compared to the other isolates (*P* < 0.001). Also, antimicrobial resistance pattern of *K. pneumoniae* isolates among biofilm formers and non-formers is shown in Table 4. The antimicrobial resistance among biofilm producing *K. pneumoniae* strains was found to be significantly higher than that of non-biofilm producing *K. pneumoniae* strains (*p* < 0.05). The correlation between biofilm production and antimicrobial resistance was found statistically significant (*p* < 0.05) in most antibiotics from different classes; CAZ, IPM, MEM: CIP, FEP, CRO, AN, and GM, but the correlation was not found to be significant in case of SXT and CTX.

**Table 3:** Biofilm formation in MDR and non-MDR *K. pneumoniae* isolates

Clinical isolates	Biofilm model			
	None (0)	Weak ( +)	Moderate (+ +)	Strong (+ + +)
MDR	8 (11.9%)	23 (34.2%)	8 (11.9%)	20 (29.8%)
Non-MDR	17 (51.5%)	8 (24.2%)	11 (33.3%)	5 (15.2%)

**Table 4:** Antimicrobial resistance pattern of *K. pneumoniae* among biofilm producer and non-producer strains

Biofilm formation ability	Strong (n = 25)			Moderate (n = 19)			Weak (n = 31)			Non-biofilm formation (n = 25)		
Antibiotics	R No. (%)	I No. (%)	S No. (%)	R No. (%)	I No. (%)	S No (%)	RNo. (%)	I No. (%)	S No (%)	R No. (%)	I No. (%)	S No. (%)
SXT	13 (52)	–	12 (48)	11 (57.8)	1 (5.2)	7 (36.8)	12 (38.7)	3 (9.6)	16 (51.6)	16 (68)	1 (4)	8 (28)
CTX	15 (60)	–	10 (40)	12 (63.1)	2 (10.5)	5 (26.3)	14 (45.1)	4 (12.9)	13 (41.9)	9 (36)	4 (16)	12 (48)
FEP	12 (48)	3 (12)	10 (40)	10 (52.6)	1 (5.2)	8 (42.1)	11 (35.4)	1 (3.2)	19 (61.2)	9 (36)	3 (12)	13 (52)
AN	4 (16)	1 (4)	20 (80)	1 (5.2)	2 (10.5)	16 (84.2)	1 (3.2)	3 (9.6)	27 (87.09)	1 (4)	1 (4)	23 (92)
IPM	13 (52)	1 (4)	11 (44)	9 (47.3)	–	10 (52.6)	6 (19.3)	1 (3.2)	24 (77.4)	7 (28)	–	18 (72)
MEM	14 (56)	-	11 (44)	7 (36.8)	1 (5.2)	11 (57.8)	8 (25.8)	–	23 (74.1)	8 (32)	1 (4)	16 (64)
CRO	13 (52)	2 (8)	10 (40)	11 (57.8)	1 (5.2)	7 (36.8)	10 (32.2)	3 (9.6)	18 (58.06)	8 (32)	4 (16)	13 (52)
GM	8 (32)	–	17 (68)	4 (21.05)	1 (5.2)	13 (68.4)	9 (29.03)	–	22 (70.9)	3 (12)	2 (8)	20 (80)
CAZ	12 (48)	4 (16)	9 (36)	12 (63.1)	1 (5.2)	6 (31.5)	13 (41.9)	3 (9.6)	15 (48.3)	9 (36)	1 (4)	15 (60)
CIP	17 (68)	3 (12)	5 (20)	12 (63.1)	–	7 (36.8)	14 (45.1)	4 (12.9)	13 (41.9)	10 (40)	2 (8)	13 (52)

*R* resistant, *S* susceptible, *I* intermediate resistant, *SXT* Trimethoprim-sulfamethoxazole, *CTX* cefotaxime, *FEP* cefepime, *AN* amikacin, *IPM* imipenem, *MEM* meropenem, *CRO* ceftriaxone, *GM* gentamicin, *CAZ* ceftazidime, and *CIP* ciprofloxacin

### The distribution of studied genes

Based on the PCR results, the carbapenemases genes identified in this study were *bla*_VIM_ (n = 7, 7 %), *bla*_IMP_ (n = 11; 11 %), *bla*_NDM_ (n = 5; 5 %), and *bla*_OXA−48_ (n = 28, 28 %). The molecular distribution of biofilm formation genes among the isolates indicated that, the most prevalent biofilm encoded genes were *luxS* with (98 %), *treC* with (96 %) and *wza* with (34 %). Finally, the co-presence of biofilm encoded factors was as follows: *luxS*/*treC/wza* (n = 21), *luxS*/*treC* (n = 62), *luxS/wza* (n = 14), and *treC/wza* (n = 8). Also, co-presence of carbapenemase genes and biofilm genes among all tested isolates was investigated. The most common coexistence was *bla*_OXA−48_*+treC + luxS* (15 %), followed by *bla*_OXA−48_*+treC + wza* + *luxS* (5 %) and *bla*_OXA−48_+*bla*_NDM_+*bla*_VIM_*+treC + wza + luxS* (3 %). The least frequent were *bla*_OXA−48_+*bla*_VIM+_*treC + wza + luxS* (2 %), *bla*_OXA−48_*+bla*_NDM_*+treC + luxS* (1 %), *bla*_OXA−48_+*bla*_NDM_+*bla*_VIM_+*treC + luxS* (1 %), *bla*_VIM_*+treC + luxS* (1 %), and *bla*_OXA−48_*+luxS* (1 %).

## Discussion

The increasing number of resistant *K. pneumoniae* strains to multiple antibiotics is a major challenge in medical centers worldwide. Heidary et al. (2018), in a systematic review and meta-analysis article, showed that there is a relatively high prevalence of drug resistant *K. pneumoniae* isolates in Iran [18]. Based on their review, the highest rate of resistance among the *K. pneumoniae* isolates was observed against ampicillin (82.2 %), aztreonam (55.4 %), and nitrofurantoin (54.5 %); while in the present study, 52, 51, 43 , and 43 % of isolates were resistant to SXT, CTX, FEP, and CRO, respectively. Khamesipour et al. (2016) indicated widespread resistance to CRO (41.1 %), SXT (36.7 %), AN (32.2 %), FEP (34.4 %) and GM (26.7 %) [19]. As well as, in the study of Moghadas et al. (2018), the antibiotic resistance rates were as follows: IPM (7.5 %), CIP (16.1 %), SXT (32.9 %), FEP (34.1 %), AN (36.4 %), and CAZ (42.7%) [20]. In contrast to our study, where we found  67%; 89.5 % of *K. pneumoniae* isolates were MDR in the study conducted by Hou et al. (2015) [21]. This discrepancy may be related to geographic distance, antimicrobial-prescribing patterns in hospitals and level of hygiene. According to the results, in DDST, of 35 IPM-resistant *K. pneumoniae* isolates, 74.3 % were MBL-positive. The prevalence of *bla*_VIM_, *bla*_IMP_, *bla*_NDM_, and *bla*_OXA−48_ was 7 %, 11 %, 5 %, and 28 %, respectively. In contrast to the present study, Carroll et al. (2013) reported that all isolates in their study were negative for *bla*_VIM_, *bla*_IMP_, and *bla*_SPM_ genes [22]. An interesting point in this research project was the presence of *bla*_*NDM*_ gene. The presence of *bla*_*NDM*_ gene in *K. pneumoniae* was first reported by Shahcheraghi et al. [23]. According to Fallah et al. [24] _NDM_ is an MBL-encoding gene, which was newly recognized and described from New Delhi, India, for the first time followed by other areas such as Pakistan. Due to the close proximity of these countries with our country and a large amount of travelling between the countries, as well as the ease of resistance transfer among microorganisms leads us to postulate that our isolates are likely have the same gene [24]. In the study of Seifi et al. of 94 *K. pneumoniae* isolates, 33 % formed fully established biofilms, 52.1 % were categorized as moderately biofilm-producing, 8.5 % formed weak biofilms, and 6.4 % were non-biofilm-producers [25]. Li et al. suggested that the expression of different adhesion, their cognate receptors, and exopolymeric components by individual cell types within a biofilm community can contribute to the general biofilm development. In particular, many bacteria are capable of using a quorum sensing mechanism to regulate biofilm formation and other social activities [26]. In this study, most of the biofilm producer strains were MDR. Our data revealed that 75 % of *K. pneumoniae* were biofilm-producing isolates. These data are similar with the findings of Seifi et al. [24]. Zheng et al. found that biofilm formation was more pronounced among *magA* (K1), *aero+*, *rmpA+*, *rmpA2+*, *allS+*, *wcaG+*, and *iutA +* isolates than in isolates which were negative for these virulence factors [17]. Wu et al. [11] concluded that *treC* and *sugE* affect biofilm formation by modulating capsular polysaccharide (CPS) production. The importance of *treC* in gastrointestinal tract colonization suggests that biofilm formation contributes to the establishment and persistence of *K. pneumoniae* infection. In agreement with Seifi et al. and Boisvert et al. strong-biofilm producing phenotypes were higher in strains isolated from sputum samples compared to other specimens [25, 27]. This indicates the important role of biofilms in the survival and colonization of microbes in the lungs, causing bacterial resistance to pulmonary clearance. In addition, a previous study sound that *luxS* was shown to be upregulated in biofilm-grown XDR *K. pneumoniae* strains [12]. Notably, in our study the *luxS* gene was detected in about 98 % of the tested isolates. Using a rat model of middle ear challenge, Yadav et al. demonstrated that a functional defect in LuxS, leading to the reduced colonization capability of pneumococci in vivo [28]. Our results exhibited that resistance to antimicrobial agents was higher in *K. pneumoniae* which is biofilm producer than non-biofilm producer. This finding is confirmed by de Subramanianet al. [29] explaining that biofilm positive uropathogens showed a high resistance rates to nalidixic acid, ampicillin, cephotaxime and trimethoprim﻿-sulfamethoxazole compared to non-biofilm producer strains, also, 80 % of the biofilm-former isolates obtained from patients demonstrated the MDR phenotype. In agreement with our results are the recent observations by Yang et al. [30] who did find a significant association between antimicrobial resistance and biofilm production in clinical isolates of *K. pneumoniae*. Nirwati et al. [8] conducted a study to recognize the drug resistance profile and biofilm-producing capability of *K. pneumoniae* isolated from clinical samples. In line with our study, a remarkable rate of their isolates were biofilm producers. They stated drug resistance was greater in *K. pneumoniae* which is biofilm producer than non-biofilm producer. However, in contrast to our results, Hassan et al. concluded that the susceptible isolates to antibiotics tend to form stronger biofilms compared with the resistant strains [31]. This discrepancy suggests that the tend to form biofilm may be an important factor in the survival of non-resistant strains. Outcomes from our investigation should be interpreted with caution, because of the disk diffusion method utilized in this study, cannot be used to assess biofilm-mediated resistance mechanisms. To overcome this limitation, other potential explanations for the correlation between biofilm formation and antimicrobial resistance (e.g. faster conjugative plasmid transfer or pleiotropic role of certain regulatory genes conferring both antimicrobial resistance and increased biofilm-forming capability) [30, 32] should be assess in the future studies to clarify these mechanisms.

## Conclusions

MDR-*Kp* is becoming a serious problem in hospitals, with many strains developing resistance to most available antimicrobials. The increasing rate of MDR-*Kp* strains emphasizes the importance of choosing an appropriate antimicrobial regimen based on antibiotic susceptibility pattern. Also, the distribution of β-lactamase producing strains is an important problem due to high antimicrobial resistance rate of them, requiring the routine evaluation and changing the antibiotic stewardship according to the results. Our findings supported the role of biofilm formation in resistance to antimicrobial agents. Most of the *K. pneumoniae* strains isolated from hospitalized patients have the capacity to biofilm production. Also, the MDR-*Kp* strains tend to form stronger biofilms than the non-MDR strains in our study. Further research on the mechanisms of biofilm formation in *K. pneumoniae* will ultimately assist in the treatment of biofilm-mediated infections and in the reduction of mortality and morbidity in patients suffering from life-threatening nosocomial infections.

## Data Availability

All data generated or analyzed during this work are included in this published article. Also. the all data used to support the findings of this study are available from the corresponding author upon request.

## Abbreviations

MDR-*Kp*: Multidrug-resistant *K. pneumoniae*
*CR-Kp*: Carbapenem-resistant* K. pneumoniae*
MBLs: Metallo-β-lactamases
H2S: Hydrogen sulfide
TSB: Trypticase Soy Broth
PCR: Polymerase chain reaction
CLSI: Clinical laboratory standards institute
MDR: Multidrug-resistant
XDR: Extensively drug-resistant
PDR: Pan drug-resistant
DDST: Double‑disk synergy test
TSBG: TSB with 1% glucose
TCP: Tissue culture plate
ODc: Optical density cut-off

## References

[1] Gorrie CL, Mirceta M, Wick RR, Judd LM, Wyres KL, Thomson NR (2018). Antimicrobial resistant *Klebsiella pneumoniae* carriage and infection in specialized geriatric care wards linked to acquisition in the referring hospital. Clin Infect Dis.

[2] Simner PJ, Antar AA, Hao S, Gurtowski J, Tamma PD, Rock C (2018). Antibiotic pressure on the acquisition and loss of antibiotic resistance genes in *Klebsiella pneumoniae*. J Antimicrob Chemother.

[3] Jafari Z, Harati AA, Haeili M, Kardan-Yamchi J, Jafari S, Jabalameli F (2019). Molecular epidemiology and drug resistance pattern of carbapenem-resistant *Klebsiella pneumoniae* isolates from Iran. Microb Drug Resist.

[4] Snitkin ES, Won S, Pirani A, Lapp Z, Weinstein RA, Lolans K (2017). Integrated genomic and interfacility patient-transfer data reveal the transmission pathways of multidrug-resistant *Klebsiella pneumoniae* in a regional outbreak. Sci Transl Med.

[5] Logan LK, Weinstein RA (2017). The epidemiology of carbapenem-resistant Enterobacteriaceae: the impact and evolution of a global menace. J Infect Dis.

[6] Muggeo A, Guillard T, Klein F, Reffuveille F, François C, Babosan A (2018). Spread of *Klebsiella pneumoniae* ST395 non-susceptible to carbapenems and resistant to fluoroquinolones in North-Eastern France. J Glob Antimicrob Resist.

[7] Yu X, Zhang W, Zhao Z, Ye C, Zhou S, Wu S (2019). Molecular characterization of carbapenem-resistant *Klebsiella pneumoniae* isolates with focus on antimicrobial resistance. BMC Genom.

[8] Nirwati H, Sinanjung K, Fahrunissa F, Wijaya F, Napitupulu S, Hati VP, Hakim MS, Meliala A, Aman AT, Nuryastuti T (2019). Biofilm formation and antibiotic resistance of *Klebsiella pneumoniae* isolated from clinical samples in a tertiary care hospital, Klaten, Indonesia. BMC Proc.

[9] Vuotto C, Longo F, Balice MP, Donelli G, Varaldo PE (2014). Antibiotic resistance related to biofilm formation in *Klebsiella pneumoniae*. Pathogens.

[10] Rathore SS, Cheepurupalli L, Gangwar J, Raman T, Ramakrishnan J (2019). Biofilm of *Klebsiella pneumoniae* minimize phagocytosis and cytokine expression by macrophage cell line. BioRxiv..

[11] Wu MC, Lin TL, Hsieh PF, Yang HC, Wang JT (2011). Isolation of genes involved in biofilm formation of a *Klebsiella pneumoniae* strain causing pyogenic liver abscess. PLoS One.

[12] Vuotto C, Longo F, Pascolini C, Donelli G, Balice MP, Libori MF (2017). Biofilm formation and antibiotic resistance in *Klebsiella pneumoniae* urinary strains. J Appl Microbiol.

[13] Clinical Laboratory Standards Institute (2018). Performance standards for antimicrobial susceptibility testing. CLSI Supplement M100.

[14] Magiorakos AP, Srinivasan A, Carey RT, Carmeli Y, Falagas MT, Giske CT, Harbarth S, Hindler JT, Kahlmeter G, Olsson-Liljequist B, Paterson DT (2012). Multidrug-resistant, extensively drug-resistant and pandrug-resistant bacteria: an international expert proposal for interim standard definitions for acquired resistance. Clin Microbiol Infect..

[15] Pournajaf A, Rajabnia R, Razavi S, Solgi S, Ardebili A, Yaghoubi S (2018). Molecular characterization of carbapenem-resistant *Acinetobacter baumannii* isolated from pediatric burns patients in an Iranian hospital. Trop J Pharm Res.

[16] Christensen GD, Simpson WA, Younger JJ, Baddour LM, Barrett FF, Melton DM (1985). Adherence of coagulase-negative staphylococci to plastic tissue culture plates: a quantitative model for the adherence of staphylococci to medical devices. J Clin Microbiol.

[17] Zheng JX, Lin ZW, Chen C, Chen Z, Lin FJ, Wu Y (2018). Biofilm formation in *Klebsiella pneumoniae* bacteremia strains was found to be associated with CC23 and the presence of wcaG. Front Cell Infect Microbiol.

[18] Heidary M, Nasiri MJ, Dabiri H, Tarashi S (2018). Prevalence of drug-resistant *Klebsiella pneumoniae* in Iran: a review article. Iran J Public Health.

[19] Khamesipour F, Tajbakhsh E (2016). Analyzed the genotypic and phenotypic antibiotic resistance patterns of *Klebsiella pneumoniae* isolated from clinical samples in Iran. Biomed Res.

[20] Moghadas AJ, Kalantari F, Sarfi M, Shahhoseini S, Mirkalantari S (2018). Evaluation of virulence factors and antibiotic resistance patterns in clinical urine isolates of *Klebsiella pneumoniae* in Semnan,&nbsp;Iran. Jundishapur J Microbiol.

[21] Hou XH, Song XY, Ma XB, Zhang SY, Zhang JQ (2015). Molecular characterization of multidrug-resistant *Klebsiella pneumoniae* isolates. Braz J Microbiol.

[22] Mathers AJ, Hazen KC, Carroll J, Yeh AJ, Cox HL, Bonomo RA (2013). First clinical cases of OXA-48-producing carbapenem-resistant *Klebsiella pneumoniae* in the United States: the “menace” arrives in the new world. J Clin Microbiol.

[23] Shahcheraghi F, Nobari S, Rahmati Ghezelgeh F, Nasiri S, Owlia P, Nikbin VS (2013). First report of New Delhi metallo-beta-lactamase-1-producing *Klebsiella pneumoniae* in Iran. Microb Drug Resist.

[24] Fallah F, Noori M, Hashemi A, Goudarzi H, Karimi A, Erfanimanesh S (2014). Prevalence of blaNDM, blaPER, blaVEB, blaIMP, and blaVIM genes among *Acinetobacter baumannii* isolated from two hospitals of Tehran. Iran Scientifica.

[25] Seifi K, Kazemian H, Heidari H, Rezagholizadeh F, Saee Y, Shirvani F (2016). Evaluation of biofilm formation among *Klebsiella pneumoniae* isolates and molecular characterization by ERIC-PCR. Jundishapur J Microbiol.

[26] Li YH, Tian X (2012). Quorum sensing and bacterial social interactions in biofilms. Sensors.

[27] Boisvert AA, Cheng MP, Sheppard DC, Nguyen D (2016). Microbial biofilms in pulmonary and critical care diseases. Ann Am Thorac Soc.

[28] Sun Y, Li Y, Luo Q, Huang J, Chen J, Zhang R (2020). LuxS/AI-2 quorum sensing system in *Edwardsiella piscicida* promotes biofilm formation and pathogenicity. Infect Immun.

[29] Subramanian P, Shanmugam N, Sivaraman U, Kumar S, Selvaraj S (2012). Antiobiotic resistance pattern of biofilm-forming uropathogens isolated from catheterised patients in Pondicherry, India. Australas Med J.

[30] Yang D, Zhang Z (2008). Biofilm-forming *Klebsiella pneumoniae* strains have greater likelihood of producing extended-spectrum β-lactamases. J Hosp Infect.

[31] Hassan PA, Khider AK (2019). Correlation of biofilm formation and antibiotic resistance among clinical and soil isolates of *Acinetobacter baumannii* in Iraq. Acta Microbiol Immunol Hung..

[32] Hennequin C, Robin F, Cabrolier N, Bonnet R, Forestier C (2012). Characterization of a DHA-1-producing *Klebsiella pneumoniae* strain involved in an outbreak and role of the AmpR regulator in virulence. Antimicrob Agents Chemother.

